# Genetic Diversity and Phylogenetic Evolution of Tibetan Sheep Based on mtDNA D-Loop Sequences

**DOI:** 10.1371/journal.pone.0159308

**Published:** 2016-07-27

**Authors:** Jianbin Liu, Xuezhi Ding, Yufeng Zeng, Yaojing Yue, Xian Guo, Tingting Guo, Min Chu, Fan Wang, Jilong Han, Ruilin Feng, Xiaoping Sun, Chune Niu, Bohui Yang, Jian Guo, Chao Yuan

**Affiliations:** 1 Lanzhou Institute of Husbandry and Pharmaceutical Sciences of the Chinese Academy of Agricultural Sciences, Jiangouyan Street, Lanzhou, China; 2 Sheep Breeding Engineering Technology Research Center of Chinese Academy of Agricultural Sciences, Jiangouyan Street, Lanzhou, China; 3 China Agricultural Veterinarian Biology Science and Technology Co. Ltd, Xujiaping, Lanzhou, China; Sichuan University, CHINA

## Abstract

The molecular and population genetic evidence of the phylogenetic status of the Tibetan sheep (*Ovis aries*) is not well understood, and little is known about this species’ genetic diversity. This knowledge gap is partly due to the difficulty of sample collection. This is the first work to address this question. Here, the genetic diversity and phylogenetic relationship of 636 individual Tibetan sheep from fifteen populations were assessed using 642 complete sequences of the mitochondrial DNA D-loop. Samples were collected from the Qinghai-Tibetan Plateau area in China, and reference data were obtained from the six reference breed sequences available in GenBank. The length of the sequences varied considerably, between 1031 and 1259 bp. The haplotype diversity and nucleotide diversity were 0.992±0.010 and 0.019±0.001, respectively. The average number of nucleotide differences was 19.635. The mean nucleotide composition of the 350 haplotypes was 32.961% A, 29.708% T, 22.892% C, 14.439% G, 62.669% A+T, and 37.331% G+C. Phylogenetic analysis showed that all four previously defined haplogroups (A, B, C, and D) were found in the 636 individuals of the fifteen Tibetan sheep populations but that only the D haplogroup was found in Linzhou sheep. Further, the clustering analysis divided the fifteen Tibetan sheep populations into at least two clusters. The estimation of the demographic parameters from the mismatch analyses showed that haplogroups A, B, and C had at least one demographic expansion in Tibetan sheep. These results contribute to the knowledge of Tibetan sheep populations and will help inform future conservation programs about the Tibetan sheep native to the Qinghai-Tibetan Plateau.

## Introduction

Tibetan sheep play agricultural, economic, cultural, and even religious roles in the Qinghai-Tibetan Plateau areas in China and provide meat, wool, and pelts for the local people [1]. The Qinghai-Tibetan Plateau areas are also rich in Tibetan sheep genetic resources, with approximately 17 indigenous sheep populations [2]. Most indigenous Tibetan sheep are not only adapted to their local environment but are also considered important genetic resources and are thus one of the major components of agro-animal husbandry societies. However, most indigenous Tibetan sheep populations are composed of relatively small numbers of individuals, and many populations have been in steady decline over the last 30 years [3]. The climate and landforms of the Qinghai-Tibetan Plateau areas are different from other areas of China. Traffic from other parts of China is blocked; thus, the Tibetan sheep are rarely influenced by external populations. These populations may now be on the verge of extinction and may ultimately be lost, given the rapid destruction of their ecological environment, the continuing introduction of modern commercial Tibetan sheep populations, and the ongoing lack of effective conservation methods [4]. To date, the genetic diversity, phylogenetic relationship, and maternal origin of the Qinghai-Tibetan Plateau populations remain uncertain and controversial.

The study of mitochondrial DNA (mtDNA) polymorphisms has proven to be tremendously useful for elucidating the molecular phylogeny of various species [5–8] due to the extremely low rate of recombination of mtDNA, its maternal lineage heredity and its relatively faster substitution rate than nuclear DNA [9]. In particular, the control region (CR), also called the displacement-loop region (D-loop) is the main noncoding regulatory region for the transcription and replication of mtDNA. One very useful approach for investigating the history and phylogenic relationships of modern domestic animals is therefore based on mtDNA sequence analysis. The variability and structure of the mtDNA control region makes it possible to describe the genetic polymorphisms and maternal origin of Tibetan sheep, mainly because mtDNA displays a simple maternal inheritance without recombination and with a relatively rapid rate of evolution [10]. The even higher substitution rate in the CR, compared with the heterogeneity rate in the other parts of mtDNA, can be used to optimally characterize intraspecific and interspecific genetic diversity [11–15].

Here, we present an investigation into the mtDNA D-loop variability observed in Tibetan sheep indigenous to the Qinghai-Tibetan Plateau areas. We aimed to increase the number of Tibetan sheep samples by including six available reference genomes from GenBank for our population genetic and phylogenetic analysis of the fifteen Tibetan sheep populations based on the complete mtDNA control region. Our results provide insight into the genetic diversity, phylogenetic evolution, and maternal origin of Tibetan sheep for the conservation and improved management of sheep genetic resources.

## Materials and Methods

### Ethic statement

We declare that we have no financial or personal relationships with other people or organizations that can inappropriately influence our work, and there is no professional or other personal interest of any nature or kind in any product, service and/or company that could be construed as influencing the position.

### Sample collection

**Fig 1:**
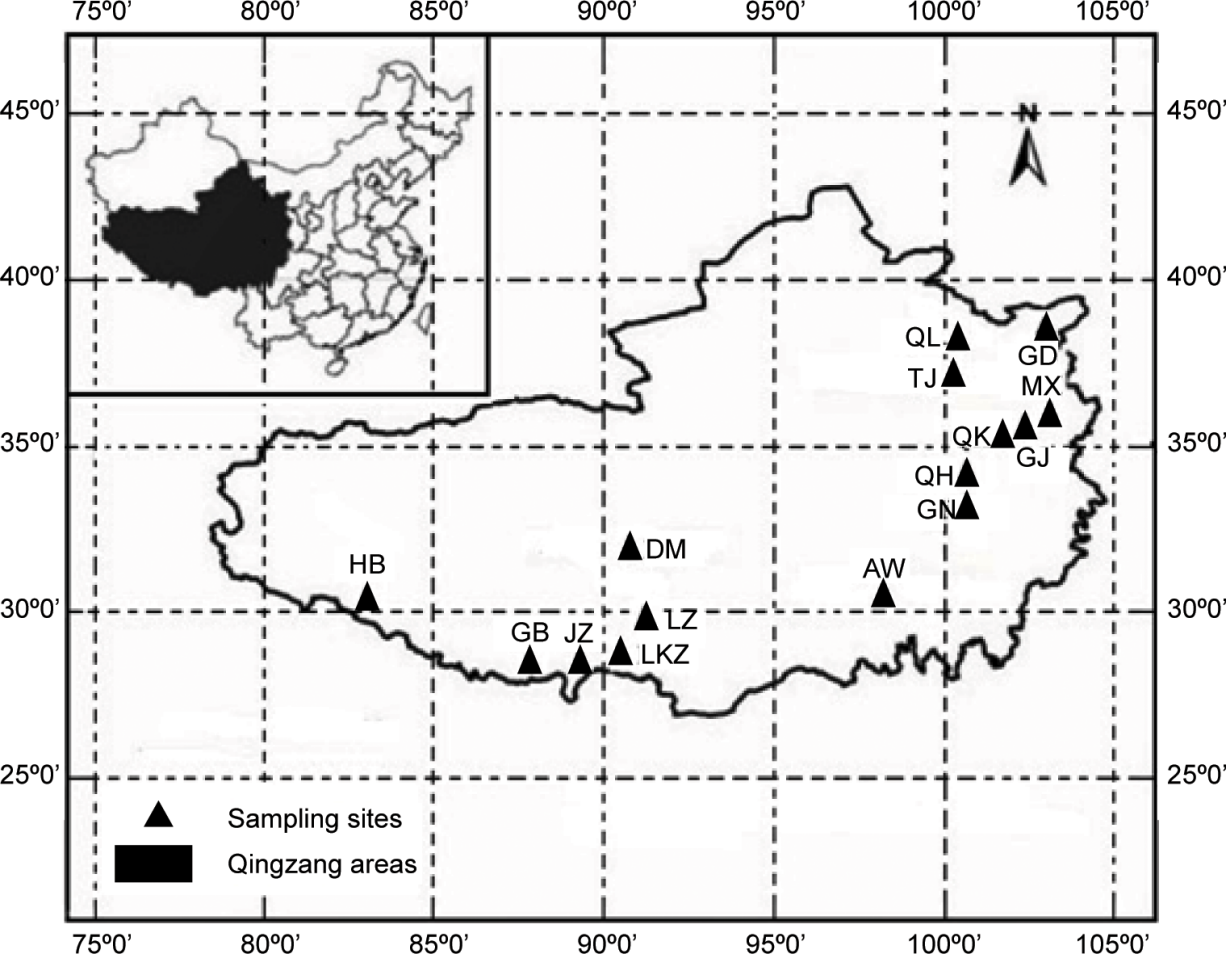
Geographical Locations of the 15 Indigenous Tibetan Sheep Populations Sampled from the Qinghai-Tibetan Plateau Area. The black area in the inset indicates the Qinghai-Tibetan Plateau area; the black triangles indicate the sampling sites within the plateau area (enlarged). The sampling locations of the specific populations are shown in Table 1.

Ten milliliters of blood was collected from the jugular vein of each animal. From the 10 mL samples, 2 mL samples were quickly frozen in liquid nitrogen and stored at -80°C for genomic DNA extraction, as described previously [16]. The total DNA was extracted from the blood using the saturated salt method [17]. The extracted DNA was quantified spectrophotometrically and adjusted to 50 ng/μL. The blood samples were collected from 636 sheep living in the Qinghai-Tibetan Plateau areas in China. The sampled individuals belonged to the fifteen Tibetan sheep populations that are distributed across Qinghai Province (Guide Black Fur sheep, n = 39; Qilian White Tibetan sheep, n = 44; Tianjun White Tibetan sheep, n = 64; Qinghai Oula sheep, n = 44), Gansu Province (Minxian Black Fur sheep, n = 67; Ganjia sheep, n = 58; Qiaoke sheep, n = 71; Gannan Oula sheep, n = 52), and the Tibet Autonomous Region (Langkazi sheep, n = 10; Jiangzi sheep, n = 46; Gangba sheep, n = 85; Huoba sheep, n = 34; Duoma sheep, n = 8; Awang sheep, n = 5; Linzhou sheep, n = 9). The sampling information (population code, sample number, altitude, longitude and latitude, accession number, sampling location, and geographical location) for the fifteen indigenous Tibetan sheep populations is shown in Table 1 and Fig 1. This study did not involve endangered or protected Tibetan sheep populations. All experimental and sampling procedures were approved by the Institutional Animal Care and Use Committee, Lanzhou Institute of Husbandry and Pharmaceutical Sciences, Peoples Republic of China. All samples were collected with the permission of the animal owners.

**Table 1:** Sampling information for the 15 indigenous Tibetan sheep populations.

Population	population code	Sample number	Altitude (m)	Longitude and latitude	Accession number	Sampling location
**Guide Black Fur sheep**	GD	39	3100	N:38°61′152″E:103°32′160″	KP228119-KP228157	Senduo Town, Guinan County, Hainan Tibetan Autonomous State, Qinghai Province
**Qilian White Tibetan sheep**	QL	44	3540	N:42°20′178″E:116°64′618″	KP228549-KP228592	Qilian Town, Qilian County, Delingha City, Mongolian Autonomous State, Qinghai Province
**Tianjun White Tibetan sheep**	TJ	64	3217	N:42°18′158″E:116°42′210″	KP228593-KP228656	Shengge Countryside, Tianjun County, Delingha City, Mongolian Autonomous State, Qinghai Province
**Qinghai Oula sheep**	QH	44	3630	N:34°16′433″E:101°32′141″	KP228434-KP228477	Jianke Village, Kesheng Town, Henan Mongolian Autonomous County, Qinghai Province
**Minxian Black Fur sheep**	MX	67	3180	N:36°54′048″E:103°94′107″	KP228367-KP228433	Taizi Village, Qingshui Town, Minxian County, Dingxi City, Gansu Province
**Ganjia sheep**	GJ	58	3022	N:35°32′049″E:102°40′802″	KP228158-KP228215	Xike Village, Ganjia Town, Xiahe County, Gannan Tibetan Autonomous State, Gansu Province
**Qiaoke sheep**	QK	71	3410	N:35°42′106″E:102°42′210″	KP228478-KP228548	Waeryi Village, Qihama Town, Maqu County, Gannan Tibetan Autonomous State, Gansu Province
**Gannan Oula sheep**	GN	52	3616	N:33°51′312″E:101°52′424″	KP228216-KP228267	Daerqing Administrative Village, Oula Town, Maqu County, Gannan Tibetan Autonomous State, Gansu Province
**Langkazi sheep**	LKZ	10	4459	N:28°58′951″E:090°23′757″	KP228348-KP228357	Kexi Village, Langkazi Town, Langkazi County, Shannan Territory of Tibet Autonomous Region
**Jiangzi sheep**	JZ	46	4398	N:28°55′113″E:089°47′692″	KP228302-KP228347	Reding Village, Cheren Town, Jiangzi County, Shannan Territory of Tibet Autonomous Region
**Gangba sheep**	GB	85	4403	N:28°15′281″E:088°24′787″	KP228034-KP228118	Yulie Village, Gangba Town, Gangba County, Rikaze Territory of Tibet Autonomous Region
**Huoba sheep**	HB	34	4614	N:30°13′822″E:083°00′249″	KP228268-KP228301	Rima Village, Huoba Town, Zhongba County, Rikaze Territory of Tibet Autonomous Region
**Duoma sheep**	DM	8	4780	N:29°48′609″E:091°36′191″	KP228026-KP228033	Sixth Village, Maqu Town, Anduo County, Naqu Territory of Tibet Autonomous Region
**Awang sheep**	AW	5	4643	N:30°12′101″E:098°63′098″	KP228021-KP228025	Ayi Third Village, Awang Town, Gongjue County, Changdou Territory of Tibet Autonomous Region
**Linzhou sheep**	LZ	9	4292	N:29°09′121″E:091°25′063″	KP228358-KP228366	Tanggu Village, Tanggu Town, Linzhou County, Tibet Autonomous Region

### Data collection

To achieve good coverage of the tested populations, a dataset of six referenced breeds was completed using the six submitted sequences containing the *Ovis aries*, *Ovis vignei*, and *Ovis ammon* mtDNA D-loops for the six individuals in GenBank (Table A in S1 File). These six breeds were from six international geographic regions and included *Omusimon*, *Ovignei*, *Oammon*, *OasiaA*, *OeuropeB*, and *Omexic*. The GenBank accession numbers for these reference sequences are AY091487, AY091490, AJ238300, AF039578 (haplogroup A), AF039577 (haplogroup B), and AY582801, respectively [10, 18, 19].

### Polymerase chain reaction and nucleotide sequencing

One pair of polymerase chain reaction (PCR) primers and sequencing primers was designed based on the 5' and 3' conserved flanking sequences of the complete mtDNA D-loop using the Primer Premier 5.0 software [20] and synthesized by BGI Shenzhen Technology Co., Ltd. (Shenzhen, China). The nucleotide sequences of forward primer CsumF was 5'-GGCTGGGACCAAACCTAT-3', and the nucleotide sequence of reverse primer CsumR was 5'-GAACAACCAACCTCCCTAAG-3'. PCR was performed in a thermal cycler (Mastercycler gradient, Eppendorf, Germany) with a total reaction volume of approximately 30 μL, containing 2 μL genomic DNA (10 ng/μL), 3 μL (3 pM) each primer, 3 μL 10×Ex Taq reaction buffer, 2 μL dNTP (2.5 mM), 0.2 μL Taq DNA polymerase (5 μL/U) (TaKaRa, China), and 16.8 μL ddH_2_O. The PCR conditions were as follows: initial denaturation for 5 min at 94°C, 36 cycles of denaturation at 94°C for 30 s, annealing at 56°C for 30 s, and extension at 72°C for 1.5 min. The final extension step was followed by a 10 min extension at 72°C. The PCR amplification products were subsequently stored at 12°C until use.

The amplified D-loop fragment was purified using a PCR gel extraction kit from Sangon Biotech Co., Ltd. (Shenzhen, China) and sequenced directly using a BigDye Terminator v3.1 cycle sequencing ready reaction kit (Applied Biosystems, Darmstadt, Germany) in an automatic sequencer (ABI-PRISM 3730 genetic analyzer, Applied Biosystems, CA, USA). PCR for the sequencing was performed in an automatic sequencer with a total reaction volume of approximately 5 μL containing 3 μL genomic DNA (10 ng/μL), 1 μL (3 pM) of each sequencing primer, 0.5 μL BigDye, and 0.5 μL ddH_2_O. The sequencing conditions were as follows: initial denaturation for 2 min at 95°C, 25 cycles of denaturation at 95°C for 10 s, and annealing at 51°C for 10 s. The final extension step was followed by a 190 s extension at 60°C. The PCR sequencing products were subsequently stored at 12°C until use.

### Data analysis

The sequences were arranged for multiple comparisons using Clustal Omega [21] and were aligned using ClustalW and BLAST [22]. These results were compared with other sequences obtained from GenBank. The reference sequences for tree construction were taken from the maternal lineages of each tree: haplogroup A (AF039578), haplogroup B (AF039577, AY582801, and AY091487), haplogroup E (AY091490, AJ238300). The diversity parameters, including the haplotype diversity, nucleotide diversity and average number of nucleotide differences, were estimated using DnaSP (Sequence Polymorphism Software) 5.10.01 [23]. The genetic differentiation coefficient (*G*_*ST*_), Wright’s F-statistics of subpopulation within total (*F*_*ST*_), gene flow (*N*_*m*_), molecular variance (AMOVA) test, and neutrality tests (Ewens-Watterson test, Chakraborty's test, Tajima's D test, Fu's FS test) were estimated using Arlequin version 3.5.1.2 [24]. To identify differences between the geographic regions using the AMOVA program, four groups were established. The phylogenetic and molecular evolutionary relationships, average number of nucleotide substitutions per site between populations (*D*_*xy*_), net nucleotide substitutions per site between populations (*D*_*a*_), ME phylogenetic haplotype and clustering tree, and genetic distance were assessed using Molecular Evolutionary Genetics Analysis (MEGA) version 6.0 [25]. We also sketched network and mismatch distribution graphs using the median-joining method implemented in the NETWORK version 4.6.1.2 software to assess the haplotype relationships [26].

## Results

### Polymorphic site and sequencing analysis of the complete control region

Based on the reference sequences from GenBank accession numbers (AY091487, AY091490, AJ238300, AF039578, AF039577, AY582801), all of the sequences were aligned with 1274 comparative sites (707 had gaps or missing data, and 567 had no gaps or missing data), and 350 haplotypes were obtained from the 642 sequenced individuals (636 Tibetan sheep and 6 reference sequences). The length of the sequences from the fifteen Tibetan sheep populations of 636 individuals varied considerably, between 1031 and 1259 bp, although the majority were between 1180 and 1183 bp (Table B in S1 File). A total of 196 variable sites were obtained from the sequences, including 63 singleton variable sites (62 double variants and 1 triple variant) and 133 parsimony-informative variable sites (124 double variants, 7 triple variants, and 2 quadruple variants). There were 158 transitions and 38 transversions within the 196 variable sites, of which 15 sites were found to have both transitions and transversions. The most commonly observed substitution caused a transition mutation. With the exception of the insertion or deletion of several nucleotide sites, the observed variations in the length of the mtDNA D-loop sequences of the Tibetan sheep mainly resulted from variability in the number of 75 bp tandem repeat motifs (between three and five repeats).

The nucleotide composition of all the haplotypes was 32.961% A, 29.708% T, 22.892% C, 14.439% G, 62.669% A+T, and 37.331% G+C. The A+T haplotype was substantially more common than the G+C haplotype, showing an AT bias (Table C in S1 File). The largest haplotype group (haplogroup A) consisted of 490 individuals and 259 haplotypes; the next largest haplotype groups (haplogroup B and haplogroup C) consisted of 145 individuals and 43 haplotypes (64 individuals and 43 haplotypes and 81 individuals and 47 haplotypes, respectively). The smallest haplotype group (haplogroup D) consisted of 1 individual and 1 haplotype. The number of haplotypes, individuals, and frequency detected in each Tibetan sheep population of haplotype group varied from 1 to 49, from 0 to 62, and from 0 to 0.875, respectively (Table 2). The haplotype diversity and nucleotide diversity were calculated separately for each Tibetan sheep population (Table 2) and were estimated to be 0.992±0.010 and 0.019±0.001, respectively. The values for the two parameters (haplotype diversity and nucleotide diversity) ranged from 0.900±0.161 to 1.000±0.045 and from 0.009±0.002 to 0.027±0.003, respectively, thus demonstrating the high level of genetic diversity in the fifteen Tibetan sheep populations. The nucleotide diversity value of the Linzhou sheep (0.027±0.003) and Jiangzi sheep (0.026±0.002) populations was found to be higher than that of the other 13 Tibetan sheep populations, indicating a relatively high level of diversity. Similarly, the haplotype diversity values were highest in the Langkazi sheep (1.000±0.045) and Linzhou sheep (1.000±0.056) populations and the lowest in the Awang sheep (0.900±0.161) population.

**Table 2:** Genetic diversity indices of fifteen Tibetan sheep populations.

Population	Nucleotide diversity	Haplotype diversity	No. of individuals	No. of haplotypes	Haplogroup A		Haplogroup B		Haplogroup C		Haplogroup D	
					No. of individuals	Frequency or No. of haplotypes	No. of individuals	Frequency or No. of haplotypes	No. of individuals	Frequency or No. of haplotypes	No. of individuals	Frequency or No. of haplotypes
**GD**	0.019±0.002	0.968±0.014	39	24	30	0.750/18	3	0.125/3	6	0.125/3	0	0
**QL**	0.015±0.002	0.998±0.006	44	40	38	0.875/35	2	0.050/2	4	0.075/3	0	0
**TJ**	0.013±0.002	0.991±0.005	64	44	57	0.869/38	4	0.045/2	3	0.086/3	0	0
**QH**	0.021±0.002	0.995±0.007	44	37	32	0.703/26	7	0.162/6	5	0.135/5	0	0
**MX**	0.015±0.002	0.932±0.018	67	25	56	0.600/15	5	0.200/5	6	0.200/5	0	0
**GJ**	0.019±0.002	0.994±0.005	58	46	45	0.740/34	7	0.130/6	6	0.130/6	0	0
**QK**	0.021±0.002	0.986±0.005	71	44	53	0.705/31	9	0.159/7	9	0.136/6	0	0
**GN**	0.021±0.002	0.986±0.007	52	37	35	0.595/22	7	0.162/6	10	0.243/9	0	0
**LKZ**	0.023±0.003	1.000±0.045	10	10	8	0.800/8	1	0.100/1	1	0.100/1	0	0
**JZ**	0.026±0.002	0.945±0.024	46	27	25	0.630/17	8	0.222/6	13	0.148/4	0	0
**GB**	0.020±0.002	0.995±0.003	85	66	62	0.742/49	10	0.106/7	12	0.152/10	0	0
**HB**	0.016±0.002	0.989±0.010	34	28	29	0.821/23	1	0.036/1	4	0.143/4	0	0
**DM**	0.017±0.003	0.964±0.077	8	7	7	0.857/6	0	0	1	0.143/1	0	0
**AW**	0.009±0.002	0.900±0.161	5	4	5	1.000/4	0	0	0	0	0	0
**LZ**	0.027±0.003	1.000±0.056	9	9	8	0.778/7	0	0	1	0.111/1	1	0.111/1
**Total**	0.019±0.001	0.992±0.010	636	350	490	0.740/259	64	0.123/43	81	0.134/47	1	0.003/1

A total of 350 haplotypes; 98 haplotypes are shared.

### Genetic distance and average number of nucleotide differences

Table 3 presents the genetic distance and average number of nucleotide differences between and within the fifteen Tibetan sheep populations. The genetic distance values ranged from 0.009 to 0.039 within the population diagonals, and the genetic distance values ranged from 0.014 to 0.040 among populations above the diagonals. Among the Tibetan sheep populations, the genetic distance within populations reached a maximum value in Linzhou sheep and a minimum value in Awang sheep. Similarly, the genetic distance between the populations had a maximum value for Linzhou sheep and Jiangzi sheep and a minimum value for Awang sheep and Tianjun White Tibetan sheep. The average number of nucleotide differences values ranged from 10.000 to 29.806 within populations along the digital diagonal, and the average number of nucleotide difference values ranged from 10.725 to 30.986 between the populations below the diagonals. Among the Tibetan sheep populations, the average number of nucleotide differences within the populations reached its value maximum in Linzhou sheep and its minimum value in Awang sheep. Similarly, the average number of nucleotide differences between populations reached a value maximum in the Linzhou sheep and Jiangzi sheep populations and a minimum value in the Awang sheep and Tianjun White Tibetan sheep populations.

**Table 3:** Genetic distance (above the diagonals) and average number of nucleotide differences (below the diagonals) between and within fifteen Tibetan sheep populations.

Population	GD	QL	TJ	QH	MX	GJ	QK	GN	LKZ	JZ	GB	HB	DM	AW	LZ
**GD**	0.026/21.579	0.023	0.022	0.027	0.023	0.026	0.028	0.029	0.027	0.033	0.027	0.024	0.023	0.020	0.032
**QL**	17.798	0.020/16.499	0.019	0.025	0.020	0.023	0.025	0.027	0.023	0.032	0.025	0.021	0.020	0.016	0.029
**TJ**	16.934	14.965	0.017/13.658	0.024	0.019	0.022	0.024	0.026	0.022	0.032	0.024	0.020	0.019	0.014	0.028
**QH**	18.492	17.325	16.504	0.029/19.902	0.025	0.027	0.029	0.030	0.028	0.034	0.028	0.026	0.025	0.021	0.033
**MX**	19.649	16.300	15.141	17.257	0.020/18.043	0.023	0.025	0.026	0.024	0.032	0.025	0.021	0.020	0.016	0.029
**GJ**	19.696	18.182	17.170	18.649	18.325	0.026/20.149	0.027	0.029	0.026	0.034	0.027	0.024	0.023	0.019	0.032
**QK**	18.884	17.717	16.691	17.376	18.033	18.937	0.029/20.542	0.030	0.028	0.035	0.029	0.026	0.025	0.021	0.034
**GN**	20.697	20.079	19.199	20.221	20.092	21.245	19.986	0.031/23.154	0.030	0.035	0.030	0.027	0.027	0.024	0.035
**LKZ**	19.374	15.409	14.528	18.091	17.825	17.034	15.986	18.638	0.029/21.444	0.035	0.028	0.025	0.024	0.019	0.032
**JZ**	25.452	25.394	24.866	23.535	25.398	26.043	24.275	25.783	21.957	0.037/29.009	0.034	0.033	0.033	0.031	0.040
**GB**	20.334	19.307	18.359	19.930	19.488	20.700	19.701	22.497	18.198	26.131	0.029/22.250	0.026	0.026	0.021	0.034
**HB**	17.951	16.383	15.484	17.757	16.548	18.608	17.936	20.147	15.865	25.246	19.446	0.022/17.118	0.021	0.017	0.030
**DM**	17.619	13.765	12.941	17.045	15.715	15.897	14.900	17.601	17.550	21.582	17.051	14.353	0.021/16.393	0.016	0.029
**AW**	17.831	12.055	10.725	14.586	15.442	14.417	14.332	17.208	16.02	23.209	15.892	12.665	14.275	0.009/10.000	0.026
**LZ**	23.966	22.663	21.528	22.876	22.919	24.602	23.203	25.808	20.411	30.986	25.421	22.944	19.36139	19.044	0.039/29.806

### Genetic differentiation

To examine the genetic differentiation between the fifteen Tibetan sheep populations, we calculated Wright’s F-statistics of subpopulation within total (*F*_*ST*_) and genetic differentiation coefficient (*G*_*ST*_) (Table 4). We also calculated the gene flow (*N*_*m*_) (Table D in S1 File), the average number of nucleotide substitutions per site (*D*_*xy*_), and the number of net nucleotide substitutions per site (*D*_*a*_) among the fifteen studied Tibetan sheep populations (Table E in S1 File). Estimates for the pairwise *F*_*ST*_ values (above diagonals) are given in Table 4. The *F*_*ST*_ values ranged from -0.046 to 0.237. Duoma sheep and Langkazi sheep had the closest pairwise *F*_*ST*_ value (*F*_*ST*_ = -0.046) among the fifteen Tibetan sheep populations. Awang sheep were more distantly related to Jiangzi sheep than they were to the other Tibetan sheep populations. All *F*_*ST*_ values were smaller than 0.25, indicating that significant genetic differentiation has not occurred among the fifteen Tibetan sheep populations. The results show that the *F*_*ST*_ values between Tibetan sheep in decreasing order were 14 (Minxian Black Fur sheep), 13 (Guide Black Fur sheep and Jiangzi sheep), 12 (Qiaoke sheep), 10 (Gangba sheep, Gannan Oula sheep and Tianjun White Tibetan sheep), 9 (Gangjia sheep, Huoba sheep, and Qinghai Oula sheep), 7 (Qilian White Tibetan sheep), 4 (Langkazi sheep and Linzhou sheep), 3 (Duoma sheep), and 1 (Awang sheep). The distribution of the fifteen Tibetan sheep populations varied according to their *F*_*ST*_ values (*P*<0.05, or *P*<0.01). The *G*_*ST*_ values ranged from 0.001 to 0.047 (Table 4). The *G*_*ST*_ value between the Langkazi sheep and Linzhou sheep was the smallest (*G*_*ST*_ = 0.001), and the *G*_*ST*_ value was the largest (*G*_*ST*_ = 0.047) (Jiangzi sheep and Awang sheep, Minxian Black Fur sheep and Awang sheep, respectively). The mean *G*_*ST*_ was 0.018, which indicates that most of the genetic diversity occurred within populations and that 1.762% of the total population differentiation came from intrapopulation, whereas the remaining 98.238% came from differences among individuals in each population. Thus, the gene divergence between the populations was very low. The result of the variation observed among and within the 15 Tibetan sheep populations was not differentiation.

**Table 4:** Estimates of pairwise *F*_*ST*_ values (above the diagonals) and *G*_*ST*_ values (below the diagonals) between fifteen Tibetan sheep populations.

Population	GD	QL	TJ	QH	MX	GJ	QK	GN	LKZ	JZ	GB	HB	DM	AW	LZ
**GD**	**——**	0.005*	0.027*	-0.001*	0.007*	-0.002*	0.003*	0.003*	-0.023*	0.056**	0.001**	-0.009*	-0.013*	0.115	-0.012*
**QL**	0.006	**——**	0.002*	0.011	0.001*	-0.001	0.009*	0.028**	-0.037	0.104**	0.010*	-0.016	-0.023	0.050	-0.022
**TJ**	0.011	0.004	**——**	0.037*	0.016*	0.016*	0.034**	0.057**	-0.021	0.148**	0.038*	0.006*	-0.014	0.065	-0.004
**QH**	0.006	0.002	0.004	**——**	0.020*	-0.008*	-0.009*	-0.009*	-0.028	0.032**	-0.009*	0.010*	0.011	0.098	-0.001
**MX**	0.024	0.018	0.015	0.016	**——**	0.010*	0.021*	0.033**	-0.022*	0.108**	0.022*	-0.005*	-0.008**	0.092*	-0.006*
**GJ**	0.010	0.001	0.006	0.003	0.022	**——**	-0.005*	0.002*	-0.032	0.062**	-0.003*	-0.001*	-0.005	0.079	-0.015
**QK**	0.009	0.005	0.006	0.003	0.022	0.005	**——**	-0.001*	-0.034*	0.044**	-0.006*	0.008*	0.007	0.102	-0.013*
**GN**	0.009	0.003	0.004	0.002	0.018	0.005	0.005	**——**	-0.020	0.014*	-0.003*	0.022*	0.016	0.141	-0.006
**LKZ**	0.015	0.011	0.015	0.013	0.025	0.015	0.017	0.013	**——**	0.039*	-0.031	-0.034	-0.046	0.019	-0.045
**JZ**	0.021	0.014	0.017	0.014	0.031	0.016	0.018	0.017	0.021	**——**	0.037**	0.093**	0.093**	0.237	0.051*
**GB**	0.008	0.002	0.004	0.002	0.017	0.004	0.003	0.003	0.017	0.015	**——**	0.007*	0.004	0.099	-0.008
**HB**	0.010	0.002	0.007	0.004	0.022	0.003	0.006	0.006	0.010	0.014	0.005	**——**	-0.029	0.072	-0.023
**DM**	0.023	0.022	0.025	0.020	0.029	0.024	0.025	0.022	0.010	0.028	0.025	0.020	**——**	0.076	-0.020
**AW**	0.043	0.042	0.044	0.040	0.047	0.044	0.045	0.042	0.030	0.047	0.044	0.042	0.025	**——**	0.050
**LZ**	0.016	0.014	0.018	0.013	0.023	0.018	0.019	0.015	0.001	0.022	0.019	0.013	0.002	0.019	**——**

*F*_*ST*_ = Wright’s F-statistics of a subpopulation within total population; *G*_*ST*_ = genetic differentiation coefficient. The *F*_*ST*_ and *G*_*ST*_ values were each calculated with 1023 permutations.

*Significant at *P* value < 0.05

** Significant at *P* value = 0.000.

Table D in S1 File presents the *N*_*m*_ of the sequence values and haplotype values between the fifteen Tibetan sheep populations. The *N*_*m*_ of sequences values ranged from -731.043 to 495.657, demonstrating that gene exchange was either extremely frequent or extremely rare between the fifteen Tibetan sheep populations. The *N*_*m*_ of the sequence values between the Gannan Oula sheep and Qiaoke sheep was the smallest (*N*_*m*_ = -731.043), and the *N*_*m*_ of the sequence values between the Minxian Black Fur sheep and Qilian White Tibetan sheep was the largest (*N*_*m*_ = 495.657). The mean *N*_*m*_ of the sequences was -9.593, implying a relatively distant relationship. The *N*_*m*_ of the haplotype values ranged from 5.041 to 177.660. Notably, the *N*_*m*_ between Qilian White Tibetan sheep and Ganjia sheep was 35.24 times greater than the *N*_*m*_ between Jiangzi sheep and Awang sheep. The *N*_*m*_ of the haplotype values between the Jiangzi sheep and Awang sheep was the smallest (*N*_*m*_ = 5.041), and the *N*_*m*_ of the haplotype values between the Ganjia sheep and Qilian White Tibetan sheep was the largest (*N*_*m*_ = 177.660). The mean haplotype *N*_*m*_ was 22.594, indicating that gene flow did not occur between the populations in the past.

Table E in S1 File shows the *D*_*xy*_ and *D*_*a*_ values among the fifteen Tibetan sheep populations. The *D*_*xy*_ values ranged from -0.0011 to 0.0050. The *D*_*xy*_ value between Langkazi sheep and Linzhou sheep was the smallest (*D*_*xy*_ = -0.0011), and the *D*_*xy*_ value between the Jiangzi sheep and Awang sheep was the largest (*D*_*xy*_ = 0.0050). The mean *D*_*xy*_ was 0.001, indicating that a low average number of nucleotide substitutions occurred per site between the fifteen Tibetan sheep populations. The *D*_*a*_ values were in the range of 0.010–0.028. The mean *D*_*a*_ was 0.019. Similarly, the number of net nucleotide substitutions per site between populations of the fifteen Tibetan sheep populations was highest in the Jiangzi sheep and Linzhou sheep (*D*_*a*_ = 0.028) and lowest in the Tianjun White Tibetan sheep and Awang sheep (*D*_*a*_ = 0.010).

### Phylogenetic relationship

**Fig 2:**
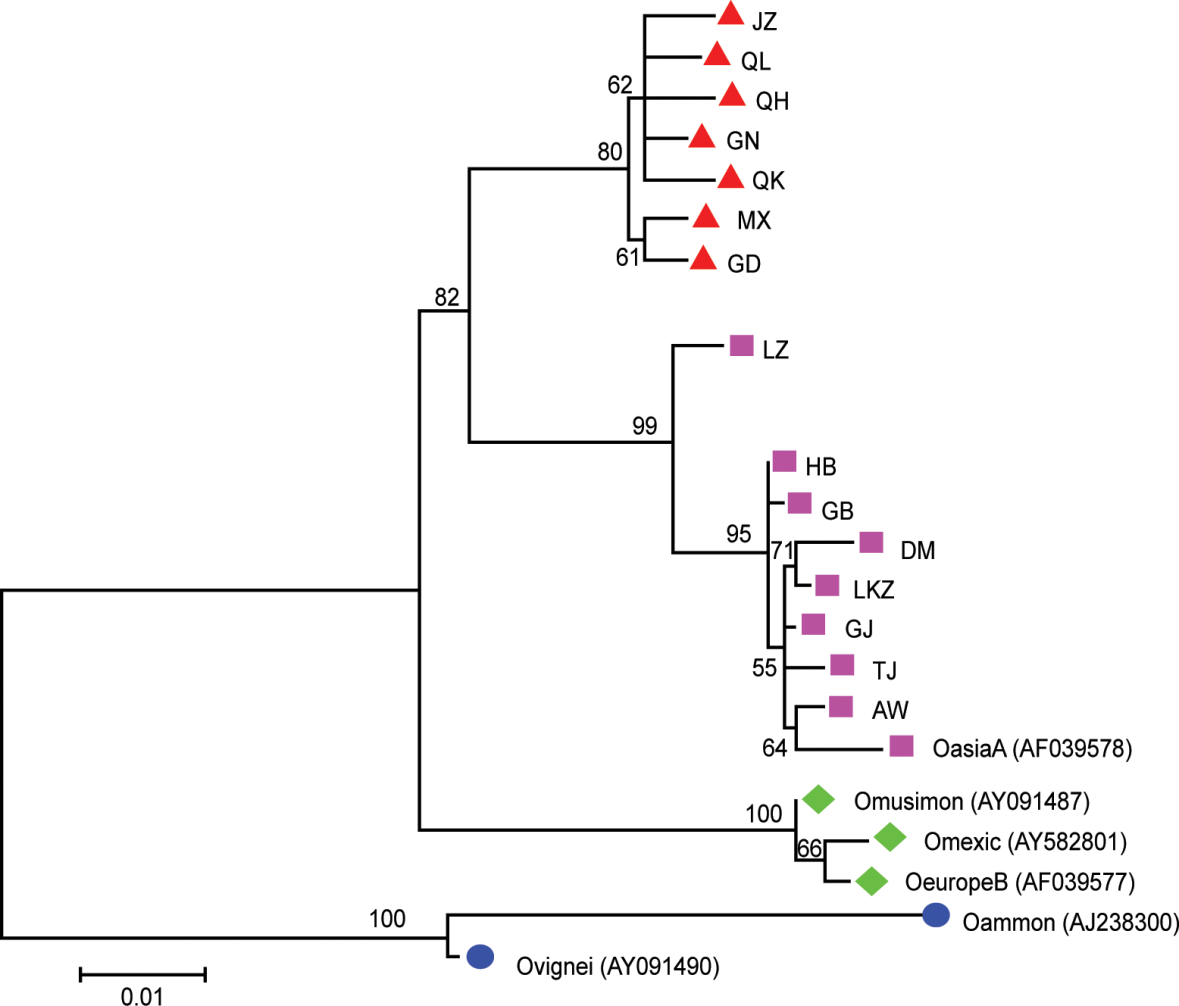
A Neighbor-joining Phylogenetic Tree of the 21 Populations based on 642 Sequences of mtDNA D-loop. The distances were computed using the Maximum Composite Likelihood method and are in the units of the number of base substitutions per site.

**Fig 3:**
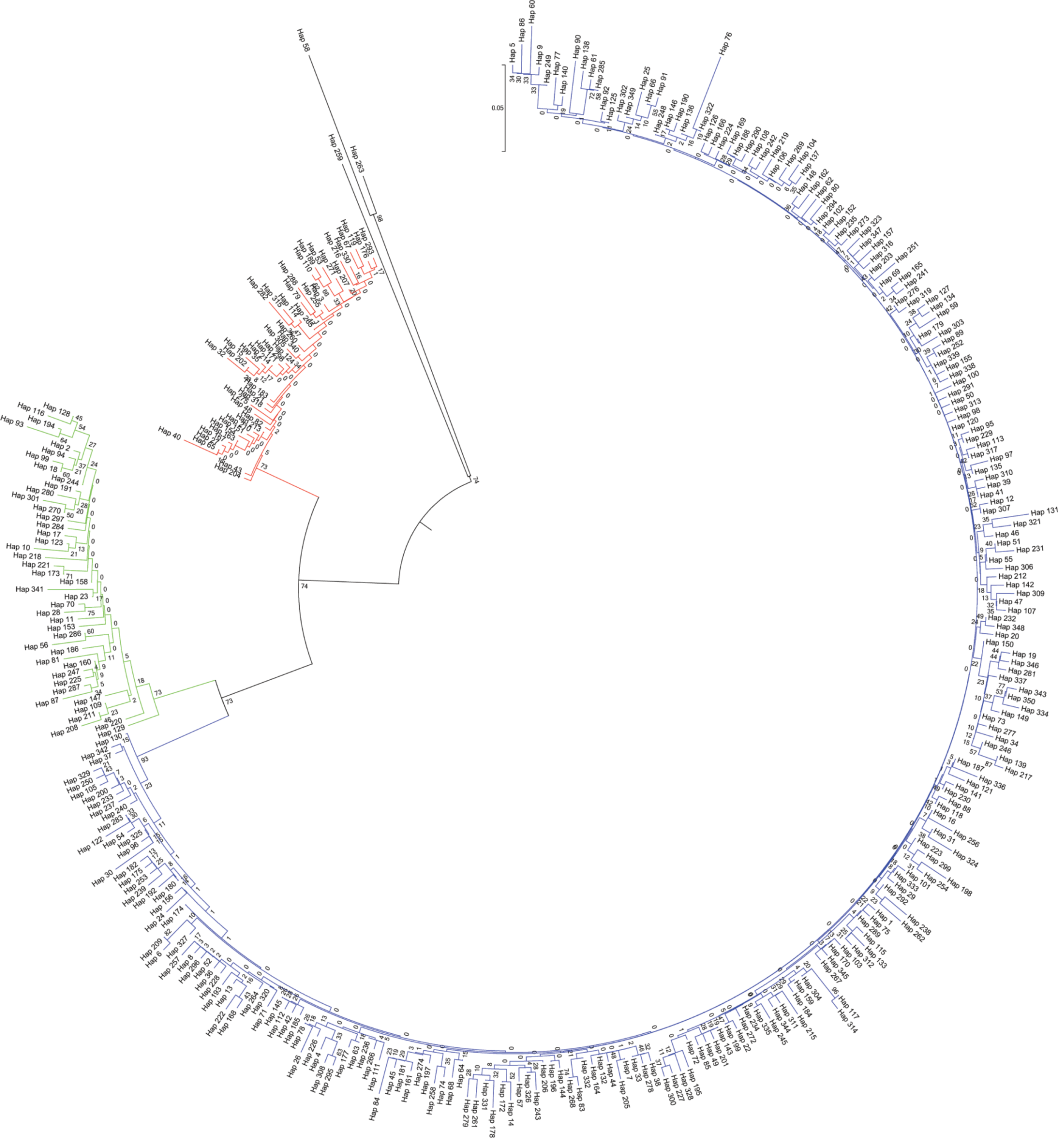
The ME phylogenetic tree of 350 haplotypes. The ME phylogenetic tree show that the 350 haplotypes and 636 sequences of Tibetan sheep populations and six reference breeds fall into five distinct clusters: haplogroup A, haplogroup B, haplogroup C, haplogroup D (Hap 259 of LZ 03) and haplogroup E (*Omusimon* and *Ovignei*), respectively. Haplogroups for individuals defined by the entire haplotypes are shaded in blue (haplogroup A), green (haplogroup B), and red (haplogroup C).

To extend our knowledge of the phylogenetic relationship of the fifteen Tibetan sheep populations, a phylogenetic tree was constructed using minimum evolution (ME), neighbor joining using the Maximum Composite Likelihood method (Fig 2) and an unweighted pair-group method with arithmetic means (UPGMA) dendrogram based on the complete mtDNA D-loop sequences of 642 individuals (Fig A, Fig B, and Fig C in S1 File) and 350 haplotypes (Fig 3) from fifteen Tibetan sheep populations and six reference breeds. The six methods produced nearly consistent topological structures and similar support levels; therefore, only the ME tree is presented (Figs 2 and 3). According to the ME tree, NJ tree, UPGMA tree, and median-joining network dendrogram (Fig D in S1 File), we determined four distinct cluster haplogroups: A (Fig A in S1 File), B (Fig B in S1 File), C (Fig C in S1 File), and D. Of the 350 haplotypes, there was no common haplotype identified in all of the Tibetan sheep populations; 98 haplotypes were shared, and 252 haplotypes were singletons, including 38 in Gangba sheep, 33 in Ganjia sheep, 28 in Tianjun White Tibetan sheep, and 24 in Qinghai Oula sheep. The leading haplotype (Hap 39) was found in 39 individuals. The next most common haplotype was Hap 42, composed of 19 individuals, and the remaining nine haplotypes were composed of seven to 10 individuals. Haplotype 42 was composed of Jiangzi sheep, Minxian Black Fur sheep, Qilian White Tibetan sheep and Tianjun White Tibetan sheep. Haplotype 4 was composed of fourteen of the Tibetan sheep populations, excluding Langkazi sheep, and showed close clustering. The majorities of the 490 individuals were grouped in haplogroup A (Fig A in S1 File), followed by haplogroups B (Fig B in S1 File) (64) and C (Fig C in S1 File) (81); however, only one animal from the Linzhou sheep (LZ03) belonged to haplogroup D. The Duoma sheep were composed of two haplogroups, the Awang sheep were composed of one haplogroup, and the remaining 13 Tibetan sheep populations were composed of three haplogroups (Table 2). The four references breeds—*OasiaA*, *OeuropeB*, *Omusimon*, and *Omexic*—belonged to haplogroups A and B. The other two reference breeds—*Omusimon* and *Ovignei*—clustered within a group (Fig 3 and Fig D in S1 File). Further, the genetic distance between populations was analyzed using the Maximum Composite Likelihood method and are in the units of the number of base substitutions per site (Fig 2). More specifically, the neighbor-joining phylogenetic tree of the 642 sequences of the mtDNA D-loop based on units of the number of base substitutions per site effectively divided the15 indigenous Tibetan sheep populations and six reference breeds into four groups. *Oammon* and *Ovignei* were genetically distinct and were the first to separate. The 15 indigenous Tibetan sheep populations and four reference breeds were then divided into three sub-clusters. The first cluster included Jiangzi sheep, Qilian White Tibetan sheep, Qinghai Oula sheep, Gannan Oula sheep, Qiaoke sheep, Minxian Black Fur sheep, and Guide Black Fur sheep. The second cluster included *OasiaA*, Awang sheep, Tianjun White Tibetan sheep, Ganjia sheep, Langkazi sheep, Duoma sheep, Gangba sheep, Huoba sheep, and Linzhou sheep. The third cluster included *Omexic*, *Oeuro*reB, and *Omusimon*. An analysis of molecular variance (AMOVA) was conducted, and the results are shown in Table F in S1 File. The AMOVA revealed a variation of 4.46% among the populations and of 95.54% within the populations; this finding was significant at *P*<0.05. The *F*_*ST*_ was 0.045, which indicated that 4.5% of the total genetic variation was due to population differences, and the remaining 95.5% came from differences among individuals in each population.

### Population expansions

**Fig 4:**
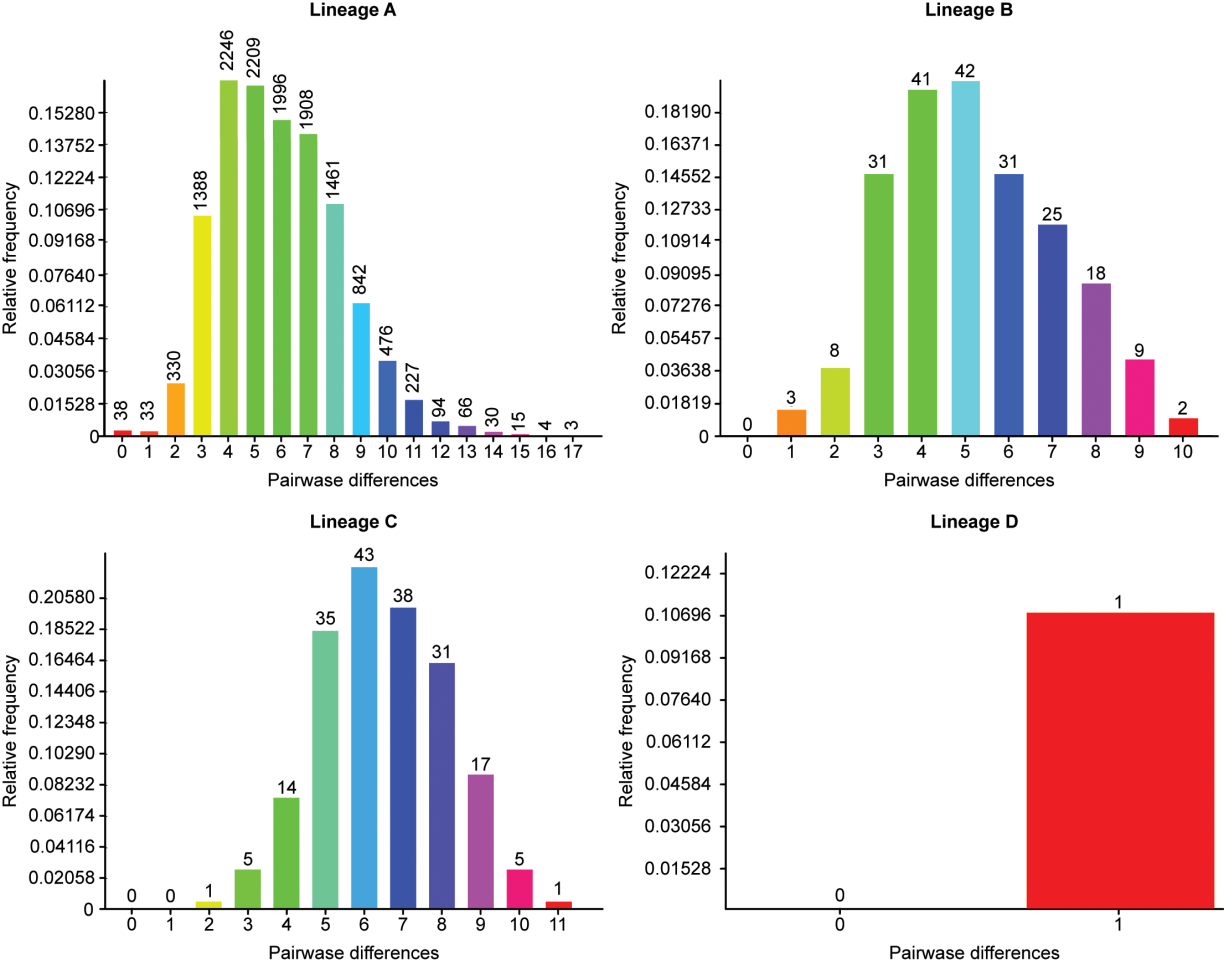
The Mismatch Distribution of Complete Dataset of Four Lineages of the Fifteen Tibetan Sheep Populations. The results were summarized in four lineages of the mtDNA types of the fifteen Tibetan sheep populations on the Qinghai-Tibetan Plateau areas showed that there was at least one demographic expansion.

Because the sample size for most of the populations was more than 30 individuals, the detection of population expansion was performed at the individual population level (data not shown) and in all haplotype sequences. The mismatch distribution analysis of the complete dataset (lineages A, B, C, D, and fifteen Tibetan sheep populations of mtDNA D-loop) is shown in Fig 4 and Fig E in S1 File. Neutrality tests (Ewens-Watterson test, Chakraborty's test, Tajima's D test, Fu's FS test) were used to detect population expansion (Table G in S1 File). The charts of the mismatch distribution for the samples of the fifteen Tibetan sheep populations and the total samples were multimodal. However, the mismatch distribution for Linzhou sheep was a unimodal function. The mismatch distribution of the complete dataset showed that there were two major peaks, with maximum values at 4 and 27 pairwise differences and two smaller peaks at 45 and 51 pairwise differences (Fig E in S1 File). These results suggest that at least two expansion events occurred during the population demographic history of the Tibetan sheep population. The mismatch distribution analysis revealed a unimodal bell-shaped distribution of pairwise sequence differences in lineages A, B and C, but that of the lineage D was a sampling function. Mismatch analysis of lineages A, B and C suggested that a single population expansion event occurred in the demographic history of Tibetan sheep populations. The complete dataset of fifteen Tibetan sheep populations did not produce a significantly negative Ewens-Watterson test, whereas Chakraborty's neutrality test of Jiangzi sheep was significant (12.629, *p* = 0.034), and Tajima's D neutrality of Tianjun White Tibetan sheep test was also significant (-0.466, *p* = 0.020). Fu's *F*_*S*_ value was -7.484 for the fifteen Tibetan sheep populations, of which Ganan Oula sheep, Qiaoke sheep, Huoba sheep, Gangba sheep, Ganjia sheep, Qinghai Oula sheep, Qilian White Tibetan sheep, and Tianjun White Tibetan sheep were highly significant (*p*<0.01 or *p*<0.001). This finding suggests the occurrence of two expansion events in the demographic history of the fifteen Tibetan sheep populations. This result is consistent with a demographic model showing two large and sudden expansions, as inferred from the mismatch distribution.

## Discussion

### High mtDNA D-loop diversity of Tibetan sheep populations

The haplotype diversity and nucleotide diversity of the total individuals were 0.992±0.010 and 0.019±0.001, respectively. The fifteen Tibetan sheep populations in our study showed a high level of haplotype and nucleotide diversity. This finding is consistent with archeological data and other genetic diversity studies [15,27–29], but the haplotype diversity found here was higher than that found in a previous study [30], and the nucleotide diversity found here was lower than that found in a previous study [4]. These results indicate a relatively higher level of genetic diversity in the fifteen Tibetan sheep populations compared with other sheep populations [1, 4, 31]. For example, the haplotype diversity and nucleotide diversity values of Turkish sheep breeds distributed in a Turkish population were 0.950±0.011 and 0.014±0.001 [31]. However, according to Walsh’s work on the required sample size for the diagnosis of conservation units [32], a sample of 59 individuals is necessary to reject the hypothesis that individuals with unstamped (“hidden”) character states exist in the population size. Thus, the sample size necessary to reject a hidden state frequency of 0.05 is 56 when sampling from a finite population of 500 individuals. Our genetic diversity estimation is therefore a precise reflection of Tibetan sheep due to the large sample size used in this study. For the Linzhou sheep, Langkazi sheep, Huoba sheep, Qinghai Oula sheep, Guide Black Fur sheep, Tianjun White Tibetan sheep, Ganjia sheep, Qiaoke sheep, Gangba sheep, and Gannan Oula sheep with broad distribution, a high genetic diversity could only be observed with such a large sample size and wide collection area. However, an even higher diversity may be found if even more samples were used, and a further investigation of the genetic diversity of these fifteen Tibetan sheep populations is still worth further research. These Tibetan sheep populations experienced a genetic bottleneck during the 20th century and are classified as the most rare populations of sheep [33]. In addition, the positive Ewens-Watterson and Chakraborty's values were significantly different among the fifteen Tibetan sheep populations, suggesting a previous decline in the population size of the mtDNA D-loop diversity. This finding was consistent with the results of a previous study [33]. Such genetic diversity may be caused by an increased mutation rate in the mtDNA D-loop, the maternal effects of multiple wild ancestors, overlapping generations, the mixing of populations from different geographical locations, natural selection favoring heterozygosis or subdivision accompanied by genetic drift [30].

### Maternal origins of the Tibetan sheep populations

The sequence motifs from the 1180 bp to the 1183 bp region of the mtDNA D-loop form the basis for the four major haplogroups (A-D) in the Tibetan sheep mtDNA haplotypes. Of these groups, haplogroup D is quite rare. The Tibetan sheep haplotypes were found to belong to all four major haplogroups, although only 0.157% belonged to haplogroup D, and these sheep were exclusively from the Linzhou sheep haplotype. This finding demonstrated that Tibetan sheep populations possess abundant mtDNA diversity and therefore a widespread origin of their maternal lineages. This study revealed a significant biogeographical association of the Asian Ovis mtDNA haplotypes with haplogroup A. Furthermore, the thoroughbred Tibetan sheep has been proposed to be shared in the haplogroup A, and the contribution of Asian sheep breeds to this population has also been reported. In this study, the overall sequences of all fifteen Tibetan sheep populations, including the fourteen Tibetan sheep populations respectively other than Duoma sheep and Awang sheep, were found in the common haplogroups B and C. It is generally believed that domestic sheep have two maternal lineages (haplogroup A and haplogroup B) based on earlier mtDNA analysis [4, 10, 18, 34]. Recently, a new maternal lineage (haplogroup C) was found in Chinese domestic sheep [27, 30]. The ME phylogenetic tree and median-joining analyses in our study revealed the presence of four mtDNA haplogroups in the Tibetan sheep populations. Of these groups, the haplogroup of lineage A was predominant, and the haplogroups of lineage B and lineage C were the second most common. In this paper, the proportion of haplotypes of lineage D was 0.157%, further demonstrating that lineage D is the most rare of the mtDNA lineages. Our findings were consistent with the results of previous studies on domestic sheep breeds in China [35, 36]. Previous studies identified three mtDNA haplogroups in both China [29, 36, 37] and other countries [38, 39, 40]. The four mtDNA haplogroups of lineages A, B, C, and D found in the Tibetan sheep populations in the Qinghai-Tibetan plateau areas further supported the hypothesis of multiple maternal origins in Chinese domestic sheep.

### Genetic differentiation of Tibetan sheep populations

The *F*_*ST*_ value represents the level of genetic differentiation within a given population. Thus, there is “little differentiation” at a value of 0.05, “moderate differentiation” at values of 0.05–0.25, and “great differentiation” at values >0.25. In this study, the AMOVA analysis also revealed the distinct population of Qinghai-Tibetan Plateau areas among other Tibetan sheep populations with a significant positive variance. Gene flow (*N*_*m*_), also known as gene migration, refers to the transfer of alleles from one population to another. *N*_*m*_ haplotype values >1 and *N*_*m*_ sequences <1 indicate a poor gene exchange, such that genetic drift will result in substantial local differentiation [41, 42]. The low *G*_*ST*_ value, combined with the low *N*_*m*_ of sequences used in this study, indicate that the great differentiation mainly resulted from the independent evolution of each isolated population and substantial local differentiation caused by the genetic drift [43]. An important factor leading to this result is likely the lower effective population sizes, as the Gannan Oula sheep, Qiaoke sheep, Ganjia sheep, and Qianlian White Tibetan sheep live in canyons and valleys and therefore have a limited ability to migrate and correspondingly lower population sizes relative to the other Tibetan sheep populations. As the effective population size declines, the nucleotide substitutions have a greater probability of reaching fixation [44, 45]. In addition, the estimated divergence time (data not shown) among the fifteen Tibetan sheep populations was consistent with the Pleistocene climate fluctuations and the uplift of the Qinghai-Tibetan Plateau, indicating that known paleogeographic factors might have played important roles in the speciation of Tibetan sheep.

### Genetic relationships among the Tibetan sheep populations

Our study showed that the fifteen Tibetan sheep populations native to the Qinghai-Tibetan Plateau are clustered into four groups: 490 Tibetan sheep represent the maternal origin of the haplogroup of lineage A, 64 Tibetan sheep represent the maternal origin of the haplogroup of lineage B, 81 Tibetan sheep represent the maternal origin of the haplogroup of lineage C, and 1 Tibetan sheep represents the maternal origin of the haplogroup of lineage D. This genetic relationship displayed a high consistency with traditional classification schemes and the results of previous studies [27, 46–49]. All fifteen Tibetan sheep populations belong to four maternal origins. The genetic differentiation of the fifteen Tibetan sheep populations was mainly the result of geographic isolation, natural selection, different living conditions, and breeding history. Because Tibetan sheep are a portable food and wool resource, the commercial trade and extensive transport of sheep along human migratory paths might help account for the observed pattern by promoting genetic exchange. Other study methods, such as genetic approaches, including the degree method and the phylogenetic relationship clustering method, also indicated that indigenous sheep were the maternal origin of haplogroups A, B, C, and D [46, 48].

### Population expansion of Tibetan sheep populations

Because the sample sizes of most of the populations were less than 34 individuals, the detection of population expansion was performed at the level of the individual populations (data not shown). The mismatch distribution analysis of the complete dataset, haplogroups A, B, C, D, and fifteen Tibetan sheep populations of the mtDNA D-loop, is presented in Fig 4 and Fig E in S1 File. Neutrality tests (Ewens-Watterson test, Chakraborty's test, Tajima's D test, Fu's FS test) were used to detect population expansion (Table G in S1 File). The complete dataset of all Tibetan sheep populations had a significantly large negative Tajima's D value and *F*_*S*_ value (Tajima's D = -0.466, *p* = 0.020; *F*_*S*_ = -7.484, *p* = 0.001). This result was consistent with a demographic model showing two large and sudden expansions, as inferred from the mismatch distribution. The mismatch distribution of the complete dataset suggested that there were two major peaks with maximum values at 4 and 27 pairwise differences and two smaller peaks at 45 and 51 differences. These results suggest that at least two expansion events occurred in the population demographic history of the Tibetan sheep living on the Qinghai-Tibetan Plateau. The mismatch distribution analysis revealed a unimodal bell-shaped distribution of the pairwise sequence differences in haplogroups A, B, and C. However, the distribution of lineage D was a sambong function. Mismatch analysis of haplogroups A, B, and C suggested that single population expansion events occurred in the demographic history of the Tibetan sheep populations. This finding was similar to the previously reported results [29].

## Conclusion

China holds abundant populations of Tibetan sheep, with significant mtDNA haplotype diversity observed in the sheep of the Qinghai-Tibetan Plateau areas. Here, the large-scale mtDNA D-loop sequences analysis of fifteen Tibetan sheep populations has provided evidence for four maternal haplogroups with high diversity. Phylogenetic analysis showed that all four previously defined haplogroups (A, B, C, and D) could be identified in the 636 tested individuals of the fifteen Tibetan sheep populations, although the D haplogroup was only found in the Linzhou sheep. The estimation of demographic parameters from the mismatch analyses shows that haplogroups A, B, and C had at least one demographic expansion in the Tibetan sheep of the Qinghai-Tibetan Plateau areas.

## Supporting Information

S1 File The UPGMA phylogenetic tree show that the 490 sequences of 15 Tibetan sheep populations (Fig A). The UPGMA phylogenetic tree show that the 64 sequences of 12 Tibetan sheep populations (Fig B). The UPGMA phylogenetic tree show that the 81 sequences of 14 Tibetan sheep populations (Fig C). Median-joining networks for the mtDNA D-loop in the control region show that 636 sequences of 15 Tibetan sheep populations and six reference breeds fall into five distinct cluster haplogroup A, haplogroup B, haplogroup C, haplogroup D and haplogroup E, respectively. The majorities of the 490 individuals were grouped in haplogroup A, followed by haplogroup B (64) and C (81); however, only one animal from LZ03 belonged to haplogroup D. The AW population was composed of one haplogroup, the DM population was composed of two haplogroups, and the remaining 13 Tibetan sheep populations were composed of three haplogroups (Fig D). The mismatch distribution of the complete dataset of the mtDNA types of Tibetan sheep of the four lineages on the Qinghai-Tibetan Plateau areas showed that there were two major peaks, with maximum values at 4 and 27 pairwise differences and two smaller peaks at 45 and 51 pairwise differences (Fig E). Mitochondrial genomes of the 6 reference breeds included in the phylogenetic analyses in this study (Table A). The length of the complete mtDNA D-loop sequence in fifteen Tibetan sheep populations (Table B). Base pair composition of mtDNA D-loop of fifteen Tibetan sheep populations (Table C). Gene flow (Nm) of the sequence (above the diagonals) and Nm of the haplotype (below the diagonals) between fifteen Tibetan sheep populations (Table D). Dxy (the average number of nuc. subs. per site between populations) (above the diagonals) and Da (the number of net nuc. subs. per site between populations) (below the diagonals) of the difference in the number of nucleotides per site differences between fifteen Tibetan sheep populations (Table E). Hierarchical analysis of the molecular variance (AMOVA) of the D-loop region of mtDNA for fifteen Tibetan sheep populations (Table F). Neutrality tests for fifteen Tibetan sheep populations (Table G).
(XLSX)
